# Inequalities in psychological distress before and during the COVID-19 pandemic among UK adults: Kishan Patel

**DOI:** 10.1093/eurpub/ckac129.745

**Published:** 2022-10-25

**Authors:** K Patel, E Robertson, ASF Kwong, GJ Griffith, K Willan, MJ Green, P Patalay, D Porteous, SV Katikreddi

**Affiliations:** 1 MRC Unit For Lifelong Health and Ageing, UCL, London, UK; 2 Social and Public Health Sciences Unit, University of Glasgow, Glasgow, UK; 3 Division of Psychiatry, University of Edinburgh, Edinburgh, UK; 4 MRC Integrative Epidemiology Unit, University of Bristol, Bristol, UK; 5 Population Health Sciences, University of Bristol, Bristol, UK; 6 Bradford Institute for Health Research, Bradford Teaching Hospitals NHS Foundation Trust, Bradford, UK; 7 Centre for Longitudinal Studies, UCL, London, UK; 8 Institute of Genetics and Cancer, University of Edinburgh, Edinburgh, UK; 9 Centre for Medical Information, University of Edinburgh, Edinburgh, UK

## Abstract

**Background:**

Evidence about how population mental health has evolved from before and over the COVID-19 pandemic remains mixed, with impacts on mental health inequalities being unclear. We investigated changes in mental health and sociodemographic inequalities from before and across the first year of the pandemic.

**Methods:**

Data from 11 UK longitudinal population-based studies with pre-pandemic measures of psychological distress were analysed, estimates pooled, and stratified by age, sex, ethnicity, country and lone household status. Trends in the prevalence of poor mental health were assessed before the pandemic (TP0) and across the pandemic at three time periods (initial lockdown (TP1), easing of restrictions (TP2), and a subsequent lockdown (TP3)).

**Results:**

In total, 49,993 adult participants were analysed across the 11 cohort studies. There was an overall worsening in mental health from pre-pandemic scores across all three pandemic timepoints, (TP1 Standardised Mean Difference: 0.15 (95% CI: 0.06 - 0.25); TP2 SMD: 0.18 (0.09 - 0.27); TP3 SMD: 0.21 (0.10 - 0.32)) with no evidence of improvement during the period of eased lockdown restrictions in summer 2020. Changes from pre-pandemic psychological distress were higher in females during the pandemic (TP3 SMD: 0.23 (0.11 - 0.35)), amongst those with degree-level education (TP3 SMD: 0.26 (0.14 - 0.38)), and adults aged 25-44 years. We did not find evidence of changes in distress differing by ethnicity, lone household status or UK nation.

**Conclusions:**

The substantial deterioration in mental health seen in the UK during the first lockdown did not reverse when lockdown lifted and a sustained worsening was observed across the pandemic. Mental health declines have been unequal across the population and these results have implications for policy, including the need for specific investment for support for those most affected to mitigate the effects of the pandemic and measures to reduce inequalities within these specific groups.

**Key messages:**

• A sustained deterioration in mental health was observed from before the start of the COVID-19 pandemic, and did not recover when social restrictions were eased.

• Deterioration in mental health varied by sociodemographic factors, namely age, sex, and education, and highlights a need for improved mental health care provision to minimise widening inequalities.

